# Supplementary material for: Touch receptor end-organ innervation and function require sensory neuron expression of the transcription factor Meis2
Source: eLife. 2024 Feb 22;12:RP89287. doi: 10.7554/eLife.89287 (PMC10942617; doi:10.7554/eLife.89287)
Supplement: Figure 1—source data 1. — Table recapitulating different Catwalk two-paw analysis parameters in 3-month-old female mice. Several recordings were performed for each mouse. Only sequences when mice showed a constant and straight locomotion with an average speed between 25 and 55 cm s-1 were selected for analysis. Student’s t-test analysis showed no significant differences for any of the parameters. [file elife-89287-fig1-data1.docx]

Figure 1 Supplementary Table 1 : Cat walk analysis

|  | WT (n=6) | | Ilset1^Cre/+^Meis2^LoxP/LoxP^ (n=7) | | *t-test* | *t-test* |
| --- | --- | --- | --- | --- | --- | --- |
| Limb | Forelimb | Hindlimb | Forelimb | Hindlimb | *Forelimb* | *Hindlimb* |
| Average speed (cm.s^-1^) | 39.49±4.42 | | 39.44±3.72 | |  |  |
| Stand (s) | 0,112±0,017 | 0,103±0,018 | 0,104±0,015 | 0,098±0,014 | *0,680* | *0,836* |
| Stand Index | -9,736±1,967 | -18,143±2,613 | -9,972±0,509 | -18,352±0,742 | *0,863* | *0,788* |
| Max Contact At (%) | 35,6±1,7 | 30,1±1,3 | 39,3±2,4 | 30,1±2,6 | *0,227* | *0,828* |
| Max Contact Area (cm²) | 0,352±0,031 | 0,352±0,011 | 0,364±0,026 | 0,370±0,028 | *0,937* | *0,397* |
| Max Contact Max Intensity | 218,0±0,6 | 226,1±0,5 | 219,0±1,2 | 226,7±1,6 | *0,842* | *0,865* |
| Max Contact Mean Intensity | 156,9±0,5 | 164,0±0,6 | 156,2±0,8 | 163,8±2,6 | *0,382* | *0,821* |
| Print Length (cm) | 0,962±0,033 | 0,857±0,018 | 1,002±0,044 | 0,971±0,054 | *0,670* | *0,060* |
| Print Width (cm) | 0,763±0,023 | 0,744±0,019 | 0,763±0,017 | 0,757±0,026 | *0,965* | *0,622* |
| Print Area (cm²) | 0,440±0,033 | 0,398±0,013 | 0,468±0,035 | 0,441±0,039 | *0,710* | *0,235* |
| Max Intensity At (%) | 17,0±2,9 | 65,2±2,8 | 23,0±1,1 | 67,8±4,2 | *0,226* | *0,603* |
| Max Intensity | 225,8±1,4 | 237,2±1,0 | 229,5±2,0 | 238,1±1,7 | *0,136* | *0,858* |
| Min Intensity | 103,0±0,9 | 105,4±1,0 | 105,0±0,8 | 107,0±1,2 | *0,139* | *0,241* |
| Mean Intensity | 165,8±1,1 | 173,6±1,3 | 166,0±1,5 | 172,9±2,9 | *0,755* | *0,929* |
| Swing (s) | 0,102±0,009 | 0,115±0,009 | 0,098±0,004 | 0,106±0,003 | *0,744* | *0,528* |
| Swing Speed (cm/s) | 81,0±8,3 | 71,7±7,7 | 89,2±5,0 | 81,9±5,0 | *0,632* | *0,473* |
| Stride Length (cm) | 7,79±0,41 | 7,80±0,38 | 8,45±0,40 | 8,45±0,41 | *0,435* | *0,439* |
| Step Cycle (s) | 0,213±0,026 | 0,214±0,027 | 0,194±0,007 | 0,195±0,007 | *0,661* | *0,662* |
| Duty Cycle (%) | 50,96±1,96 | 44,74±2,23 | 49,54±1,32 | 45,20±0,94 | *0,764* | *0,717* |
| Toe Spread (cm) | 0,558±0,029 | 0,632±0,017 | 0,507±0,023 | 0,626±0,032 | *0,198* | *0,924* |
| Intermediate Toe Spread (cm) | 0,430±0,050 | 0,398±0,020 | 0,550±0,013 | 0,415±0,023 | *0,114* | *0,995* |
| Manual Print Length (cm) | 0,822±0,034 | 0,729±0,026 | 0,845±0,045 | 0,832±0,055 | *0,888* | *0,093* |
| Paw Angle Body Axis (°) | 3,574±2,155 | 7,471±1,720 | 1,140±0,109 | 7,274±2,076 | *0,399* | *0,727* |
| Paw Angle Movement Vector | 5,182±2,341 | 4,920±1,221 | 5,016±1,147 | 3,735±1,587 | *0,991* | *0,960* |
| Single Stance (s) | 0,095±0,012 | 0,093±0,014 | 0,090±0,003 | 0,088±0,003 | *0,818* | *0,967* |
| Initial Dual Stance (s) | 0,008±0,003 | 0,005±0,003 | 0,004±0,001 | 0,00±0,001 | *0,372* | *0,365* |
| Terminal Dual Stance (s) | 0,009±0,003 | 0,006±0,003 | 0,004±0,001 | 0,002±0,001 | *0,279* | *0,317* |
| Body Speed (cm/s) | 36,275±5,619 | 35,296±5,597 | 39,803±2,571 | 38,306±2,252 | *0,787* | *0,820* |
| Body Speed Variation (%) | 19,484±2,633 | 19,599±2,386 | 18,686±2,399 | 20,481±2,643 | *0,935* | *0,548* |
